# Abstract of the Proceedings of the Medical Society of the State of New-York at Its Annual Meeting in February, 1847

**Published:** 1847-04

**Authors:** 


*Abstract of the Proceedings of the Medical Society of the State of New-York at its annual meeting in February,* 1847.—We are able to anticipate the official publication of the proceedings of the Medical Society of the State of New-York, by availing ourselves of the following abstract from the pen of Dr. Lee, editor of the New-York Journal of Medicine and the collateral Sciences, published, by permission of the Society, in the March number of that valuable Journal. Our readers, will recognize in these proceedings evidences that the Society participates in the spirit of the times.—The appointment of committees to report upon different scientific subjects will add greatly to the interest of the meetings, as well as to the character and usefulness of the Society. We omit a portion of the abstract, containing a preamble and resolutions submitted by Dr. Blatchford, of Troy, which were published in the last number of this Journal :

*Abstract of the Proceedings of the Medical Society of the state of New York, at its annual Meeting, in February,* 1847.—We were much gratified in being able to attend, for the first time, the recent annual meeting of our State Medical Society, which convened at Albany on the 2d, and continued in session until the 5th of February. The representation from different parts of the State was highly respectable ; and the entire proceedings were characterized by the utmost harmony and good feeling. Indeed, we have no recollection of ever having attended a medical convention, where such a variety of topics were discussed, sometimes of a character likely to excite controversy, or endanger conflicting interests, and where such perfect cordiality and unanimity prevailed throughout. The delegates were treated with marked respect by the Legislature, who granted their assembly-room for the delivery of the President's adress ; and during two successive evenings the members were entertained in the true Knickerbocker style, by Drs. Wing and McNaughten, whose generous hospitality will not soon be forgotten.—The following is a brief summary of the Transaction, which will soon be published officially :—

On the convening of the Society in the Capital, Feb. 2d, the President, Dr. John McCall, of Utica, read a very able adress on the subject of medical science, its present condition, with suggestions for its improvement, which was ordered to be published. Communications were then read from the following societies on the subject of medical education, viz., Onedia, Albany, and Erie. A committee was appointed, consisting of Drs. Stevens, Lee, March, Brigham, and Burwell, to report on the subject of the President's adress.—Dr. A. H. Stevens, of New York, called the attention of the Society to the subject of idiocy, and offered the following memorial to the Legislature, which was adopted :—

"The medical Society of the State, understanding that a bill is now before your honorable body, to provide for the instruction of idiots, take leave most earnestly to reccommend the object, as one in their view eminently deserving of your favorable consideration, and expresses the hope that the great State of New York will not fail to secure to herself the honor of being the first among her fellows, in undertaking this great work of benevolence."

Whereon it was resolved unanimously, that the President and Secretary sign and transmit said document to the Senate.

The Treasurer, Dr. Van Olinda, presented his Annual Report.—Dr. Davis, chairman of the committee on medical education, presented a report on that subject. An invitation was received from Professor J. McNaughten, to a social entertainment at his house in the evening, which was accepted. Reports containing a list of the number of graduates, with the terms of study, were presented from the College of Physicians and surgeons, New York, and from the Albany Medical College.

Dr. Blatchford, of Troy, offered the following resolution, which after some debate was adopted :—

" *To the Honorable the Legislature of the State of New York.*

" The memorial and petition of the Medical society of the State, respectfully sheweth, that a bill is now before your honorable body, granting aid to all the medical colleges and faculties in the State except the College of Physicians and Surgeons of New York. They ask that this institution may receive your support equally with the others, and the more especially as it has ever, and does now confer medical degrees, not in virtue of the recommendation of the medical faculty alone, but of disinterested parties, and is successfully laboring with kindred institutions, to elevate the standard of medical education, and your petitioners, as in duty bound, will ever pray."

A communication was then received from the Columbia County Medical Society, through Dr. Pruyn, delegate, being the annual adress of Dr. Bates, to said Society, which was referred to the Publishing Committee.

Dr. Stevens read a very interesting Essay on the pathology of disease and the modus operandi of noxious agents in the production of disease, for which the thanks of the Society were presented, and a copy requested for publication.

On motion of Dr. S. N. Davis, twenty delegates were chosen to represent the State Society in the National Medical Convention, to meet in Philadelphia in May next. The following were elected :—

*Southern District*—John Stearns, John W. Francis, Edward Delafield, James R. Manley, E. G. Ludlow ; *Middle District*—J. W. Sprague, J. McCall, A. Willard, N. S. Davis, P. H. Hard ; *Eastern District*—Joel A. Wing, Daniel Ayres, T. W. Blatchford, D. Clark, Morgan Snydre ; *Western District*—Maltby Strong, Alex. Thompson, L. J Teft, G. W. Bradford, Enos Barnes.

In the resolution to appoint delegates, it was expressly stated that nothing contained in it, was intended to debar the several County Societies from electing and being represented by Delegates in the Convention.

[TO BE CONCLUDED IN OUR NEXT NUMBER.]

